# The Foundry: the DNA synthesis and construction Foundry at Imperial College

**DOI:** 10.1042/BST20160007

**Published:** 2016-06-09

**Authors:** Stephen Chambers, Richard Kitney, Paul Freemont

**Affiliations:** * SynbiCITE, Imperial College, London SW7 2AZ, U.K.

**Keywords:** commercialization, engineering biology, Foundry, innovation, synthetic biology, translation

## Abstract

The establishment of a DNA synthesis and construction foundry at Imperial College in London heralds a new chapter in the development of synthetic biology to meet new global challenges. The Foundry employs the latest technology to make the process of engineering biology easier, faster and scalable. The integration of advanced software, automation and analytics allows the rapid design, build and testing of engineered organisms.

## Introduction

The publication of the Roadmap for Synthetic Biology in 2012 and its recommendations resulted in significant U.K. Government investment in synthetic biology. Over £100 million was invested through the Research Councils U.K. and Innovate U.K. to establish six new Synthetic Biology Research Centres (SBRCs: Universities of Bristol, Edinburgh, Manchester, Nottingham, Cambridge/John Innes Centre and Warwick), four Doctoral Training Centres (DTCs: Universities of Oxford, Bristol, Warwick and University College, London) and an Innovation and Knowledge Centre (IKC: SynbiCITE at Imperial College, London) to drive the commercial translation of synthetic biology research. The U.K. Government's commitment to the commercialization of synthetic biology was further reinforced with an additional £10 million investment in the Rainbow Seed Fund for synthetic biology spinouts and start-ups. One of the more innovative initiatives to come out of this investment was the creation of a U.K. DNA synthesis capability to support synthetic biology academic research and commercial translation. In total, five DNA Synthesis Foundries were established (Edinburgh Genome Foundry, Liverpool Gene Mill, MRC Laboratory of Molecular Biology, DNA Synthesis and Construction Foundry at Imperial College and The Genome Analysis Centre at Norwich Research Park).

The DNA Synthesis and Construction Foundry at Imperial College (the Foundry) is located at SynbiCITE, which operates from the Imperial Incubator. SynbiCITE as the national centre for the translation and commercialization of synthetic biology provides a number of innovation programmes to its academic and industrial partners. These innovation programmes are specifically designed to provide early-stage business support and include: education and training, funding for proof of concept and development of prototypes and business support. An important element in the innovation programmes SynbiCITE provides is access to its laboratory facilities, including–the Foundry. This access provides scientists and researchers with a maker space for the rapid translation of ideas and designs: producing a prototype or generating critical data–all geared towards advancing technology, removing the technical uncertainty and risk in the process. This facility is available to SynbiCITE's extensive network of partners including 17 U.K. universities and over 50 industrial and commercial partners, made-up of multinational companies, SMEs and start-ups.

## Focus

SynbiCITE with its specific focus on commercial translation employs the Foundry to provide a suite of state-of-the-art robotic and analytical equipment supplying end-to-end design, construction and characterization capability to its partners. Work is focused on industry-led projects with commercial or practical applications, which can benefit from the Foundry's automated higher throughput and capacity.

## Goals

1. Build a state-of-the-art DNA Synthesis and Construction Foundry. Operated as a series of modular automated workstations the Foundry provides an integrated, flexible assembly platform capable of delivering biological design automation at scale.
2. Develop the U.K. capability and infrastructure for synthetic biology. The creation and operation of standardized workflows and software for DNA synthesis, assembly and verification of large DNA assemblies (>50 kb), including chromosome synthesis at scale.
3. Support the U.K. industrial base in synthetic biology. The Foundry as part of SynbiCITE, the national IKC for Synthetic Biology, will provide access, collaboration and training opportunities to U.K. companies interested in synthetic biology.
4. Provide support and training programmes. The Foundry, in collaboration with its partners, act as a focal point for the training of researchers (both academic and industrial) in the synthesis and construction of DNA, at scale, in relation to synthetic biology applications.

## Facilities and resources

The Foundry's facilities and resources are available to all SynbiCITE academic and industrial partners–providing access to both equipment and expertise. A list of all the equipment is available at www.synbicite.com. To keep abreast of the fast pace of technology development in synthetic biology, the facilities and resources available at the Foundry will evolve to meet demand. The Foundry has continued access to the latest cutting edge technology, required to stimulate innovation in synthetic biology, is made possible through a number of special collaborations with industrial partners supplying resources and expertise.

People with the correct skills are key to the successful adoption of synthetic biology by industry. We also recognize that this new industry depends on people with the right skill-set to translate their ideas. SynbiCITE is therefore committed to sharing new approaches and discoveries in synthetic biology with its partners by providing scientific workshops and technical training on all equipment and platforms.

A major aim of the Foundry (and synthetic biology) is to make the design and engineering of biological systems systematic and standardized around a ‘design-build-test-report-learn’ cycle. The Foundry has been specifically built to support and exploit this framework.

The facilities and accompanying capabilities include:

- A range of commercial and proprietary BioCAD/CAM tools for the rapid and scalable **design** of transgenes and experiments.
- Conventional and acoustic liquid handling robotic platforms to **build** and assemble multiple DNA components in parallel. Supported by software for programming and scheduling automation.
- A suite of high-throughput analytical/**test** equipment for metadata acquisition allowing characterization and quantification across the range of biological products.
- A web-based Laboratory Information Management System (LIMS) to **report** on the tracking of the workflow for any assembly or experiment. The LIMS tracks the users’ specifications containing the product design, the entire construction sequence including replicating reagents, PCR and assembly; and the sample reagent lineage, location and availability.
- The engineering-design cycle is completed with data analytics, providing the **learn** capability with statistical interpretation and modelling which can feedback into the design loop.

The Foundry and SynbiCITE, both represent a unique public–private collaborative effort to accelerate and promote business exploitation of emerging research and technology around synthetic biology. The Foundry intends to deliver a key resource to the U.K. synthetic biology industrial and academic community seeking to commercialize their ideas and research.

## Abbreviations

IKC: Innovation and Knowledge Centre
LIMS: Laboratory Information Management System
SBRC: Synthetic Biology Research Centre

